# Pain and distress outcomes in infants and children: a systematic review

**DOI:** 10.1590/1414-431X20175984

**Published:** 2017-07-03

**Authors:** N.C.A.C. Oliveira, C.M. Gaspardo, M.B.M. Linhares

**Affiliations:** Departamento de Neurociências e Ciências do Comportamento, Faculdade de Medicina de Ribeirão Preto, Universidade de São Paulo, Ribeirão Preto, SP, Brasil

**Keywords:** Children, Development, Distress, Pain, Review

## Abstract

The aim of the present study was to systematically review the recent literature about pain and distress outcomes in children and critically analyze the methodological quality of the reports. The systematic review was based on the PRISMA statement and performed by selecting articles that are indexed in scientific databases. The methodological quality of reports was examined using STROBE statement, for observational studies, and CONSORT statement, for randomized controlled trials. The PedIMMPACT consensus was used to evaluate the psychometric quality of pain instruments. We analyzed 23 empirical studies, including 14 randomized controlled trials, seven cross-sectional studies, and two studies with cohort designs. Fourteen studies included preschool- and schoolchildren, and nine studies included infants. Regarding studies with infants, pain responses were evaluated by heart rate, crying and behavioral observation scales, and distress was evaluated only by salivary cortisol. Four-handed care and sensorial saturation interventions were used to evaluate efficacy to reduce pain and distress responses. Concerning studies with children, both pain and distress responses were evaluated by self- and hetero-reports, behavioral observation and/or physiological measures. Distraction was effective for reducing pain and distress during burn dressing changes and needle procedures, and healing touch intervention reduced distress and pain in chronic patients. All of the studies scored at least 60% in the methodological quality assessment. The pain outcomes included measures of validity that were classified as well-established by the PedIMMPACT. This systematic review gathers scientific evidence of distress-associated pain in children. Pain and distress were measured as distinct constructs, and their associations were poorly analyzed.

## Introduction

Pain constitutes a global health problem, the relief and treatment of which are recognized as a human right by health organizations, especially the World Health Organization and International Association for the Study of Pain (1). Although there has been an exponential increase in scientific studies on pain assessment and management in recent decades, pain continues to be under-assessed and under-treated in pediatric patients (2–5).

Pain is an adverse and stressful experience that can have a negative impact on child development and quality of life. Acute pain has been shown to be positively associated with distress and anxiety (6,7), and chronic pain with helplessness and depression (8,9). Acute painful procedures are a major source of distress in pediatric patients and may have long-term consequences on behavior (10), memory (11), pain perception (12), and developmental outcomes in children (13).

Previous reviews have evaluated randomized clinical trials (RCTs) with regard to the efficacy of non-pharmacological interventions for pain relief in infants (14,15) and children (16–19). One recent review analyzed studies about factors predicting anticipatory distress to painful medical procedures in children aged 0-18 years old (20). However, these reviews did not have the main propose of analyzing studies that assessed pain and distress outcomes simultaneously in infants and children specifically. A recent review also highlighted the need to improve the methodological quality of studies on the subject, allowing the development of guidelines for clinical practice (21).

Distress factors increase the risk of physiological symptoms, negative memories of pain, fear and non-cooperation in future painful procedures, as well as burdening the health system with the management of preventable emotional illnesses (22). Therefore, the aim of the present study was to systematically review the recent literature that assessed both pain and distress outcomes in children and to perform an analysis of the methodological quality of the reports.

## Material and Methods

### Databases

The systematic search strategy was based on the Preferred Reporting Items for Systematic reviews and Meta-Analysis (PRISMA) statement (23). The first author conducted the literature search, data extraction, and critical appraisal. The other authors reviewed the material. Scientific articles were selected in the PubMed, Web of Science, LILACS, and PsycINFO databases. The following combinations of key words were used for the search: *Pain* and *Stress* and *Behavior*, *Pain* and *Distress* and *Behavior*, *Pain* and *Stress* and *Development*, and *Pain* and *Distress* and *Development*.

The inclusion criteria were empirical studies that included pain and distress outcomes published in the last 5 years (from 2010 to 2015), included samples of children between 0-12 years of age, and were published in English. The exclusion criteria were: review articles, letters, editorials, commentaries, studies that evaluated pain or distress in parents and professionals, studies on pharmacological or epidemiological issues exclusively, and studies in languages other than English.

**Figure 1.**
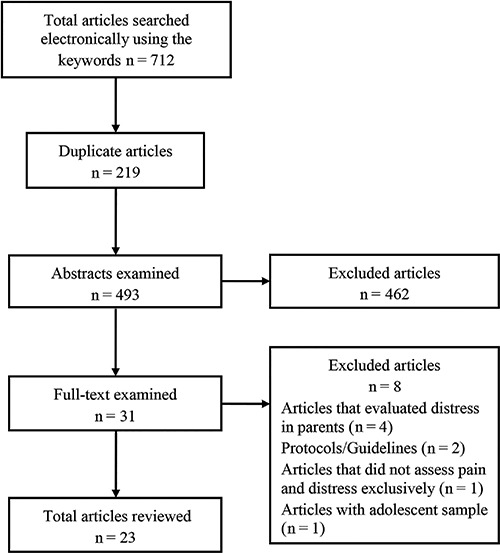
Flowchart of the search strategy used in the review.

Figure 1 summarizes the literature review process. The initial database search yielded 712 articles. Of these, 219 were duplicated in other databases. After analyzing 493 abstracts, 462 articles were eliminated according to the exclusion criteria. The major reasons for exclusion were: articles that did not assess both pain and distress outcomes (n=168), theoretical articles (n=107), articles with other age groups (n=65), articles that evaluated pain or distress in parents or professionals (n=40), and studies on pharmacological or epidemiological issues (n=35). Thirty-one full-text articles were then reviewed using the inclusion and exclusion criteria. The reasons for exclusion in this stage are shown in Figure 1. A total of 23 articles were finally selected for this systematic review.

### Data treatment

Data extraction was performed to collect information regarding the purpose of the study, theoretical framework, intervention, design, methods, analysis, and findings. The methodological quality of the studies was examined according to the following methodological guidance reports: Strengthening the Reporting of Observational studies in Epidemiology (STROBE) statement (24) for observational studies and the Consolidated Standards of Reporting Trials (CONSORT) statement (25) for RCTs. The total score for STROBE and CONSORT was calculated for each study. The maximum total score was 22 for STROBE and 37 for CONSORT. A higher percentage of items conforming to the guidelines represent higher methodological quality.

The Pediatric Initiative on Methods, Measurement, and Pain Assessment in Clinical Trials (PedIMMPACT) consensus was used to determine the psychometric quality of instruments that were used in the studies to evaluate pain (26,27). The PedIMMPACT consensus establishes criteria regarding the quality of pain assessment instruments, classifying them as well-established, approaching well-establishment, and promising, based on the validity, reliability, and measurement parameters detailing.

## Results

### Characteristics of the studies

Most of the articles were from the United States (n=9, 39%), followed by Canada (n=3, 13%), Australia (n=2, 9%), and Sweden (n=2, 9%). The other seven articles were published in different countries. With regard to the methodological designs of the studies, 14 (61%) of the articles were RCTs (27–40), 7 (30%) used a cross-sectional design (41–47) and 2 (9%) used a cohort design (48,49).

Fourteen studies (61%) included children of preschool and school age (3-12 years old) (28–30,32,34,36,38,40,41,43,44,47,48,50), and 9 (39%) included infants (0-2 years old) (31,33,35,37,39,42,45,46,49). The majority of the studies assessed acute pain during medical procedures (n=20, 87%). Only three studies (13%) evaluated chronic pain conditions (41,43,48).

Among the 23 studies analyzed, 15 (65%) used self-reported and/or hetero-reported instruments to measure pain intensity, 11 studies (48%) applied behavioral assessments of pain, and 7 studies (30%) used physiological measures. With regard to distress outcomes, 12 studies (52%) used physiological measures (e.g., salivary cortisol) to measure distress, 8 (35%) applied behavioral assessments of distress, and 6 (26%) used children's self-reports and/or caregivers' reports.

### Methodological quality of the studies

Table 1 shows the methodological quality of the studies. All conformed with at least 60% of the CONSORT and STROBE statements. Of the 14 RCTs that were analyzed according to the CONSORT statement, 6 conformed with 60-70% of the items (31,33,36,37,39,41), 1 conformed with 70-80% of the items (35), and 7 conformed with >80% of the items (28–30,32,34,38,40). These results indicate that the studies reviewed herein had high methodological quality. The nine observational studies evaluated according to the STROBE statement also demonstrated good methodological quality; 4 conformed with 60-70% of the items (46,47,48,50), 4 conformed with 70-80% of the items (42–45), and 1 conformed with >90% of the items (49).

**Table 1.** Methodological quality of the studies based on the percentage of conforming items of the CONSORT and STROBE guidelines (n=23).

Study group/References	CONSORT (%)	STROBE (%)
Randomized clinical trials		
Infants		
Mitchell et al., 2013 (39)	67%	NA
Cone et al., 2013 (31)	66%	NA
Gitto et al., 2011 (33)	62%	NA
McGowan et al., 2013 (37)	67%	NA
Preschool and school age		
Brown et al., 2014 (30)	88%	NA
Nilsson et al., 2013 (40)	84%	NA
Miller et al., 2011 (38)	87%	NA
McCarthy et al., 2014 (36)	68%	NA
Hartling et al., 2013 (34)	90%	NA
Baxter et al., 2011 (28)	84%	NA
Beran et al., 2013 (29)	84%	NA
De Jong et al., 2012 (32)	87%	NA
Wong et al., 2013 (41)	69%	NA
Cross-sectional and cohort		
Infants		
Castral et al., 2012 (42)	NA	77%
Mehler et al., 2015 (45)	NA	77%
Grunau et al., 2010 (49)	NA	94%
Schuller et al., 2012 (46)	NA	65%
Preschool and school age		
McCarthy et al., 2010 (44)	NA	77%
Connelly and Bickel, 2011 (43)	NA	74%
Telli et al., 2015 (47)	NA	67%
Smith et al., 2015 (50)	NA	68%
Zhao et al., 2015 (48)	NA	61%

NA: not applicable. CONSORT: Consolidated Standards of Reporting Trials; STROBE: Strengthening the Reporting of Observational studies in Epidemiology.

The following weaknesses were identified in the 14 RCTs according to the CONSORT statement: no identification of being a randomized trial in the title (n=11), lack of a trial registration number (n=10), no estimated effect size of the results (n=8), and no description of blinding characteristics (n=8). For the nine observational studies, the following weaknesses were identified according to the STROBE statement: no identification of the study design in the title or abstract (n=8), absence of a flowchart diagram (n=8), absence of sample size information (n=7), and no explanation of how missing data were addressed (n=7).

With regard to the psychometric quality of the pain measurements, all of the instruments that were used in the studies were classified as well-established by the PedIMMPACT consensus.

### Main findings of infant studies

*Interventions for pain and distress relief in infants*. Table 2 shows the main features and findings of the five RCTs that examined the efficacy of different types of interventions to reduce pain and distress in infants. Three studies compared nonpharmacological interventions (i.e., kangaroo care, four-handed care, and glucose administration) with standard care in a neonatal intensive care unit during endotracheal/nasopharyngeal suctioning procedures in preterm newborns (31,35,39). Two of these studies used a crossover design (31,35). The results showed that kangaroo care and glucose administration were not significantly different from standard care with regard to the reduction of pain and distress (35,39). Four-handed care was more effective than standard care in reducing pain (measured by physiological measures of heart rate and oxygen saturation) and distress outcomes in preterm newborns (39).

**Table 2.** Main findings of randomized clinical trials that assessed pain and distress responses in infants (n=5).

Sample Groups; n; GA; age	Painful procedure	Pain measure	Distress measure	Pain management	Main results
Mitchell et al., 2013 (39)					
– Kangaroo care (KC); 19; 27-30 wks; <5 days – Standard care (SC); 19; 27-30 wks; <5 days	Endotracheal or nasopharyngeal suctioning	PIPP scale	Salivary cortisol	KC *vs* SC	KC=SC for pain and distress
Cone et al., 2013 (31)					
– Four-handed care (FHC); 10; <37 wks; <7 days	Endotracheal suctioning	HR, SpO_2_	Salivary cortisol; ABSS	FHC *vs* SC	FHC <SC for pain and distress behavior (ABSS)
Ivars et al., 2012 (35)					
– Preterm glucose (PT); 11; <37 wks; <28 days – Full-term (FT); >37 wks;2 days	Nasopharyngeal suctioning	VAS, HR, SpO_2_	Salivary cortisol	PT glucose *vs* PT no glucose *vs* FT	PT glucose=PT no glucose=FT for pain and distress
Gitto et al., 2011 (33)					
– Fentanyl (FE); 50; 27-32 wks; 2 days – Facilitated tucking (FT); 50; – Sensorial saturation (SS);27-32 wks; 2 days	Heel-lance for blood collection	CRIES score	Cytokine levels	FE *vs* FT *vs* SS	FE and SS <FT for pain SS <FE for distress
McGowan et al., 2013 (37)					
– Intervention care group(simultaneous); 36; 2-6 months –Control care group (sequential); 36; 2-6 months	Immunization	MBPS	VAS distress	Simultaneous *vs* sequential administration techniques	Simultaneous <sequential for observed pain Simultaneous= sequential for distress

GA: gestational age; wks: weeks; PIPP: Premature Infant Pain Profile; HR: heart rate; SpO_2_: oxygen saturation; ABSS: Anderson Behavioral State Scale; VAS: Visual Analogue Scale; CRIES: C: crying, R: requires increased oxygen administration, I: increased vital signs, E: expression, S: sleeplessness; MBPS: Modified Behavior Pain Scale.

The other two studies evaluated different interventions during needle procedures (i.e., heel-lance for blood collection and immunization). Three types of interventions, including pharmacological (fentanyl) and nonpharmacological (facilitated tucking and sensorial saturation) management for pain and distress relief were examined in preterm newborns on postnatal day 2 (33). Fentanyl and sensorial saturation were more effective than facilitated tucking in reducing pain scores. Sensorial saturation was more effective than fentanyl in reducing distress scores. McGowan et al. (37) assessed two different needle techniques (simultaneous and sequential) that were applied during immunizations in infants. The main findings showed that the simultaneous technique was more effective than the sequential technique in reducing pain and distress scores (measured by parental report) in infants at 2-6 months of age.

*Evaluation of pain and distress outcomes in infants.* Table 3 shows the features and results of the four observational studies that evaluated needle procedures (immunization and heel puncture) in infant samples. Three of the studies performed between-group comparisons (45,46,49), and one study analyzed associations between variables (42).

**Table 3.** Main findings of cross-sectional and cohort studies on pain and distress responses in infants (n=4).

Design/Sample Groups; n; GA; mean age	Painful procedure	Pain measures	Distress measures	Main results
Mehler et al., 2015 (45)				
Cross-sectional				
– Very preterm (VPT); 61; <32 wks; 3.6 months – Late preterm (LPT); 30; ≥32 and <37 wks; 3.5 months – Full-term (FT); 30; ≥ 37 and <42 wks; 2.7 months	Immunization	BPNS, HR, withdrawal threshold	Salivary cortisol	VPT <LPT and FT for physiological (HR) and behavioral (BPNS) pain VPT <LPT and FT for pain threshold reactivity LPT >FT >VPT for distress
Grunau et al., 2010 (49)				
Cohort				
– Extremely low gestational age (ELGA); 29; ≤28 wks; 3.6 months – Very low gestational age (VLGA); 39; 29-32 wks; 4.5 months – Full-term; 32; 38-41 wks; 4.5 months	Immunization	NFCS, HR	Salivary cortisol	ELGA=VLGA=Full-term for pain ELGA and VLGA boys <Full-term boys for distress
Schuller et al., 2012 (46)				
Cross-sectional				
– Elective cesarean section (ELSC); 112; 38 wks, 3 days – Spontaneous vaginal delivery (SVD); 107; 39 wks, 3 days – Vacuum extraction (VE); 61; 28 wks, 3 days	Heel prick (at first 72 h postnatal period)	BPNS, EDIN	Salivary cortisol	VE >SVD and ELSC for pain (EDIN) VE >ELSC for distress
Castral et al., 2012 (42)				
Cross-sectional				
– Preterm infants (PT) under Kangaroo care; 42; <36 wks; 6 days	Heel puncture for blood collection	Cry; NFCS, HR	Salivary cortisol	↑ Mothers' cortisol levels before painful procedure ↑ PT cortisol levels after painful procedure and ↑ pain (NFCS) during painful procedure

GA: gestational age; wks: weeks; NFCS: Neonatal Face Coding Scale; HR: heart rate; EDIN: Echelle Douleur Inconfort Nouveau; BPNS: Bernese Pain Scale for Neonates.

Very preterm infants (infants born less than 32 weeks of gestational age) presented less pain reactivity, lower pain thresholds, and lower distress compared with full-term infants during immunization procedures (45). Grunau et al. (49) observed lower distress responses during immunization procedures in boys who were born extremely preterm or with a very low gestational age compared with boys who were born full-term and then evaluated at 4 months of age.

Distress and pain responses in newborns were evaluated during the early postpartum period according to the mode of delivery. Newborns who were delivered vaginally presented higher pain scores (measured by observational scales) and distress scores during heel puncture procedures compared with infants in the elective cesarean section group (46).

The relationship between maternal factors and pain and distress responses in preterm infants who received maternal kangaroo care during heel puncture procedures was investigated by Castral et al. (42). High levels of salivary cortisol in mothers before the heel puncture procedure were correlated with high levels of salivary cortisol in neonates after the procedure and high pain expression during the procedure.

### Main results of children studies

*Interventions for pain and distress relief in children*. Table 4 presents the characteristics and results of the nine RCTs with children samples. The first three studies in Table 4 examined different interventions during wound dressing procedures in pediatric burn patients. Interventions that were based on distraction (i.e., Ditto intervention, Multimodal Distraction, and Serious gaming) were more effective than standard care and another intervention (i.e., lollipops) in reducing pain (measured by combined tools) (30,38,40) and distress (38,40).

**Table 4.** Main findings of randomized clinical trials that evaluated pain and distress responses in children (n*=*9).

Sample Medical condition; total n; age range; Groups; n; mean/median age	Painful condition	Pain measures	Distress measures	Intervention for pain management	Main results
Brown et al., 2014 (30)					
– Pediatric burn patients; 75;4-12years – Ditto intervention; 35; 8.24 years – Standard care (SC); 40; 8.32 years	Wound dressing	FPS-R, FLACC, HR, SpO_2_	Salivary cortisol, CTSQ	Ditto intervention (preparation and distraction) *vs* SC	Ditto group <SC for pain (FPS-R, FLACC, and HR)
Nilsson et al., 2013 (40)					
– Pediatric burn patients; 60; 5-12 years – Serious gaming; 20; 8 years – Lollipops; 20; 7 years – Control group (CG); 20; 7 years	Wound dressing	CAS, FLACC	FAS	Serious gaming *vs* Lollipops *vs* CG	Serious gaming <Lollipops and CG for pain (FLACC) and distress
Miller et al., 2011 (38)					
– Pediatric burn patients; 40; 3-10 years – Multimodal distraction protocol (MMD); 20; 6.11 years – Standard distraction (SD); 20; 5.91 years	Wound dressing	Wong Baker Faces Scale (FACES), VAS, HR	FLACC	MMD *vs* SD	MMD <SD for pain and distress
McCarthy et al., 2014 (36)					
– Children with all medical diagnoses; 574; 4-10 years – Low distress risk (LDR): basic; 46; 8.4 years – Medium distress risk (MDR): basic; 121; 7.3 years; enhanced; 125; 7.3 years; professional; 79; 7.6 years – High distress risk (HDR): enhanced; 126; 7.1 years; professional; 77; 6.4 years	Intravenous insertion	Oucher scale	OSBD-R, salivary cortisol, PPQ	LDR *vs* MDR *vs* HDR Basic *vs* Enhanced *vs* Professional distraction interventions	LDR <MDR (Basic) for distress MDR <HDR (Enhanced and Professional) for distress Professional <Basic and Enhanced for distress (OSBD-R)
>Hartling et al., 2013 (34)					
– Children in pediatric emergency department; 42; 3-11 years – Music intervention; 21; 6 years – Standard care (SD); 21; 6 years	Intravenous insertion	FPS-R, HR	OSBD-R	Music *vs* SC	Music <SC for pain (FPS-R) and distress
Baxter et al., 2011 (28)					
– Children in pediatric emergency department; 81; 4-18 years - Device (Buzzy); 41; 10.10 years - Standard care (SC); 40; 9.91 years	Venipuncture	FPS-R	OSBD	Device (Buzzy) *vs* SC	Buzzy <SC for pain and distress
Beran et al., 2013 (29)					
– Children under immunization; 57; 4-9 years - Humanoid Robot; 28; 6.36 years - Control group (CG); 29; 6.66 years	Immunization	FPS-R	BAADS	Humanoid robot *vs* CG	Humanoid robot <CG for pain and distress
De Jong et al., 2012 (32)					
– Children with craniosynostosis; 59; 3-36 months - Massage with mandarin oil; 20; 10.1 months - Massage with carrier oil; 20; 11.5 months - Standard care (SC); 19; 10.8 months	Postoperative care	NRS pain, COMFORT, HR, mean arterial pressure	NRS distress	Massage with mandarin oil *vs* Massage with carrier oil *vs* SC	Massage with mandarin oil=Massage with carrier oil=SC for pain and distress
Wong et al., 2013 (41)					
– Pediatric oncology patients; 9; 3-18 years - Healing touch (HT); 6; 8.83 years - Reading/activity; 3; 7.33 years	Oncological pain	Wong Baker Faces Scale	Feeling Thermometer	HT *vs* Reading/play activity	HT <Reading/play activity for pain and distress

NRS: Numerical Rating Scale; HR: heart rate; FLACC: Face, Legs, Activity, Cry, Consolability; SC: standard care; FPS-R: Faces Pain Scale-Revised; CG: control group; CTSQ: Child Trauma Screening Questionnaire; CAS: Color Analogue Scale; FAS: Facial Affective Scale; VAS: Visual Analogue Scale; OSBD-R: Observational Scale of Behavioral Distress-Revised; PPQ: Perception of Procedures Questionnaire; BAADS: Behavioral Approach-Avoidance Distress Scale.

The other four studies evaluated different interventions during needle procedures (i.e., intravenous insertion, venipuncture, and immunization) and reported positive results for the efficacy of distractions in reducing distress and the efficacy of music, a Buzzy¯ device (MMJ Labs, USA), and a humanoid robot in relieving pain and distress compared with standard care and the control condition (28,29,34,36). All of these studies used self-reports to assess pain and behavioral observations to assess distress. McCarthy et al. (36) compared three levels of distress risk (low, medium, and high) and three levels of distraction intervention (basic, enhanced, and professional) and found that the groups with low and medium risk for distress benefited more from a distraction. Additionally, when professional distraction was applied, the level of distress was less than when basic or enhanced distraction was applied.

Only one study examined two different types of massage (with mandarin oil or carrier oil) for the relief of pain and distress under a postoperative care condition (32). No significant difference was found between these two massage techniques and standard care in children with craniosynostosis. In contrast, Wong et al. (41) evaluated two interventions for oncological pain relief in children with cancer, showing that the healing touch intervention was more effective than reading or playing activities in reducing both pain and distress responses that were measured by self-report.

*Evaluation of pain and distress outcomes in children*. Table 5 presents the characteristics and findings of five observational studies in children. Three studies assessed acute pain conditions (44,47,50), two of which evaluated needle procedures (44,50). McCarthy et al. (44) examined children’s pain and distress (measured by combined tools) during an intravenous insertion procedure with parental distraction coaching. Younger age was associated with higher pain and distress. Child impulsivity was associated with high pain intensity. A high level of parental distraction coaching was associated with less distress in children. Pain and distress during a needle procedure were also examined by Smith et al. (50), who argued that an imbalance in the apelin and endothelin systems contributed to an increase in the number of painful vaso-occlusive episodes and baseline pain in children with sickle cell disease.

**Table 5.** Main findings of cross-sectional and cohort studies on pain and distress responses in children (n*=*5).

Design/Sample Medical condition; total n; age range Group; n; mean/median age	Painful condition	Pain measures	Distress measures	Main results
McCarthy et al., 2010 (44)				
Cross-sectional				
– Children with all medical diagnoses under parental distraction; 542; 4-10 years; 6.95 years	Intravenous insertion	Oucher scale	OSBD-R, salivary cortisol, PPQ	↓ Age ↑ Pain intensity and distress (OSBD-R) ↑ Child impulsivity ↑ Pain intensity ↑ Level of parental distraction coaching ↓ Child distress (OSBD-R)
Smith et al., 2015 (50)				
Cohort				
– Children with sickle cell disease; 47; 2-18 years; 9.98 years	Venipuncture	Wong Baker Faces Scale; VAS	OSBD	Imbalance in apelin and endothelin systems ↑ Painful vaso-occlusive episodes and baseline pain
Telli et al., 2015 (47)				
Cross-sectional				
– Pediatric urology patients; 120; 3-8 years – Top-down (TD); 60; 5.2 years – Bottom-up (BU); 60; 4.9 years – Repeated voiding cystourethrography (R-VCUG); 544;.6 years	Invasive radiological procedures (VCUG and DMSA)	FPS-R	CAMPIS-R	TD=BU=R-VCUG for pain ↑ Child distress ↓ Child coping ↑ Child distress ↓ Adult coping
Connelly and Bickel, 2011 (43)				
Cross-sectional				
– Children with headache; 25; 8-17 years; 12.34 years	Headache episodes	VAS, electronic diary	Facial Affect Scale	↑ Distress intensity ↑ Headache occurrence
Zhao et al., 2015 (48)				
Cross-sectional				
– Children with cerebral palsy; 40; 1-4 years; 2.27 years – Spastic; 21; 2.38 years – Nonspastic; 19; 2.14 years	Pain during intervention programs (NDT; NMES; OT; HA and CTM)	FLACC; WRTs (pain sensitivity)	Salivary cortisol	Spastic > Non-spastic for pain in NDT intervention ↑ Distress levels in HA, NDT, NMES, and CTM posttreatment in Spastic and Non-spastic

VCUG: voiding cystourethrography; DMSA: technetium dimercaptosuccinic acid; CAMPIS-R: Child-Adult Medical Procedure Interaction Scale-Revised; OSBD: Observational Scale of Behavioral Distress; PPQ: Perception of Procedures Questionnaire; NDT: neurodevelopmental treatment; NMES: neuromuscular electrical stimulation; OT: occupational therapy; HA: head acupuncture; CTM: Chinese traditional manipulation.

Pain and distress in pediatric urology patients who were undergoing invasive radiological procedures were examined by Telly et al. (47). Three approaches (top-down, bottom-up, and repeated voiding cystourethrography) were compared during an invasive procedure, and significant differences in pain and distress outcomes were found between groups. However, a negative correlation was found between child distress and child and adult coping behavior, indicating that high child distress was associated with lower child and adult coping behavior.

Only two studies evaluated chronic pain conditions. In school-age children with headache episodes, a high level of distress predicted a higher incidence of headache (43). Children with cerebral palsy (spastic and non-spastic) were assessed during five treatment programs (acupuncture, neurodevelopmental treatment, neuromuscular electrical stimulation, Chinese traditional manipulation, and occupational therapy), which can cause some degree of pain and distress themselves (48). The children in the spastic group presented significantly higher pain than the non-spastic group, especially in the neurodevelopmental treatment group.

## Discussion

The present systematic review provides relevant considerations in children’s pain and distress research. We identified a large number of RCTs, showing a clear concern of the scientific community to design studies to test the efficacy of nonpharmacological interventions for pain relief. Previous reviews have addressed RCTs of non-pharmacological interventions for pain relief in specific age groups (14,16,18–21), without necessarily assessing pain and distress responses simultaneously. The present review found that most RCTs and observational studies had satisfactory methodological quality, conforming with more than 70% of the items of the CONSORT and STROBE statements.

With regard to methodological controls, most of the studies utilized standard care in routine settings and provided details of the painful procedures, thus contributing to a better understanding of the intervention, context, and findings. With regard to methodological care, we found that the studies used well-established measures based on the PedIMMPACT consensus, thus providing greater reliability of the studies' findings. The criteria for considering a measure as well-established were the following: validity, reliability, measurement accuracy that allows replication, and publication by different groups of researchers in peer-reviewed journals (26,27). With regard to pain outcomes, one RCT (30) used only physiological measures to assess pain in infants. Although these indicators are sensitive to pain, they should be considered complementary measures, and not necessarily pain-specific (51).

Overall, most of the studies used physiological and behavioral parameters as distress outcomes. The present review found few instruments that measured distress, especially in infants, in which salivary cortisol was the most commonly used measure. It is important to note that when a child feels threatened, there are biobehavioral reactions with physiological responses, including increase in heart rate, blood pressure, and stress hormones (e.g., cortisol) (52). However, future studies should focus on instruments that advance beyond physiological indicators, including behavioral dimensions of distress in infants.

Most studies assessing distress in pre-school and school phases used observational scales that presented well-established psychometric proprieties (53) and only four studies measured salivary cortisol (30,36,44,48). Few studies used self-reported tools to measure distress outcomes, with a highlight on the use of generic measures, such as Feeling Thermometer, Numerical Rating Scale, Visual Analogue Scale and Facial Affective Scale, which are not distress-specific (27,32,37,40,41). This demonstrates a demand for self-reported instruments that specifically measure distress outcomes in children. Moreover, the terms distress and pain are often used interchangeably. Pain and distress are indeed associated with each other through distinct constructs. Distress is described as a type of negative emotion that could interfere in painful procedures and have psychological implications in a child’s development (18,54), but it should be observed separately from other negative emotions, such as pain experience.

A larger number of studies assessing preschool- and schoolchildren rather than infants was found. There is relevant evidence showing that infants who were exposed to painful experiences at very early developmental stages present distress reactivity with a long-term developmental negative impact (11,13).

Another aspect that was highlighted in the present review is the lack of studies on chronic pain compared to studies on acute pain, which were found in a greater number. This may be related to the fact that acute pain is the main complaint of children and parents during medical procedures, and acute pain is intrinsically related to distress in pediatric settings (55,56). Efforts have been made to determine the long-term impact of acute painful procedures on child behavior and development (10,11,13), which may be associated with the development and maintenance of chronic pain later in life (12).

In conclusion, this systematic review gathers scientific evidence of distress-associated pain in children in different developmental phases, which may contribute to better care for infants and children under painful conditions. The present review has some limitations that should be addressed. First, the review period was restricted to the last 5 years. Second, the studies used different interventions, painful procedures, pain and distress outcome measures, and sample sizes, which may hinder comparisons between studies. Third, we adopted an upper limit for age that consequently excluded studies with adolescents.

Future studies should address chronic pain and develop specific instruments to evaluate distress in infants while maintaining methodological quality to improve the reliability of the findings. Finally, the pain and distress reactivity should be better examined separately, but in the same painful experience context. Furthermore, the data analysis should advance into the associations between pain and distress variables.
